# Typical course of cystinuria leading to untypical complications in pregnancy: A case report and review of literature

**DOI:** 10.3389/fmed.2023.1097442

**Published:** 2023-04-06

**Authors:** Ema Ivandic, Marjan Maric, Vesna Elvedi-Gasparovic, Margareta Fistrek Prlic, Lovro Lamot, Bojan Jelakovic, Ivana Vukovic Brinar

**Affiliations:** ^1^Department of Nephrology, Arterial, Hypertension, Dialysis and Transplantation, University Clinical Hospital Zagreb, Zagreb, Croatia; ^2^Clinic of Urology, University Clinical Hospital Zagreb, Zagreb, Croatia; ^3^Department of Obstetrics and Gynecology, University Hospital Centre, Zagreb, Croatia; ^4^Division of Pediatric Nephrology, Dialysis and Transplantation, Department of Pediatrics, University Hospital Center Zagreb, Zagreb, Croatia; ^5^School of Medicine, University of Zagreb, Zagreb, Croatia

**Keywords:** cystinuria, pregnancy, case report, chronic kidney disease, multidisciplinary approach

## Abstract

Cystinuria is a rare genetic disorder inherited by an autosomal recessive pattern which affects the transmembrane transporter for the base amino acid cystine. It has a general prevalence of 1 in 7000 with demographic variations. Patients with cystinuria have excessive urinary excretion of cystine, which can lead to the formation of stones. Up to 70% of patients will develop chronic kidney disease that can progress even to end-stage renal disease. Symptoms usually start in the first two decades of life with a typical presentation consisting of flank pain and renal colic, usually accompanied by urinary tract infection and deterioration of kidney function. Men are typically affected twice as often as women and have a more severe clinical course. Diagnosis is made by spectrophotometric analysis of the stones that are collected after spontaneous expulsion or medical intervention. Genetic testing is not mandatory but is recommended in uncertain cases or as a part of genetic counseling. Treatment consists of diet modification, alkalization of urine, and thiol-based therapies if other measures fail to prevent stone formation. In pregnancy, cystinuria with the formation of cystine stones represents a therapeutic challenge and requires a multidisciplinary approach consisting of an uro-nephrology team and a gynecologist. We present the case of a 34-year-old woman with cystinuria on whom the diagnosis was made by analysis of the expulsed stone. While her previous pregnancies were without complications, her third pregnancy was accompanied by frequent urinary tract infections, acute worsening of kidney function, and urological interventions during pregnancy due to the formation of new stones. Despite the complicated course, the pregnancy was successfully carried to term with the delivery of a healthy female child.

## Introduction

Cystinuria is a rare genetic disorder that affects the transmembrane transporter for the base amino acid cystine leading to excessive excretion of cysteine in urine that induces stone formation. Cystine transporter b^0,+^ is located in the proximal tubule. Its heterodimer is combined of two subunits—rBAT and b^0,+^ AT connected with a disulfide bridge (1). Solute carrier protein 7 (*SLC7A9*) located on chromosome 19 encodes subunit b^0,+^ AT, while solute carrier protein 3 (*SLC3A1*) located on chromosome 2 encodes for rBAT. Currently, there are more than 200 mutations connected with *SLC3A1* and more than 100 mutations associated with *SLC7A9*, but according to some authors, up to 5% of patients have cystinuria without known mutation (2). Transepithelial transporter is also responsible for the transportation of ornithine, lysine, and arginine (COLA) (3).

According to the International Cystinuria Foundation, cystinuria can be divided into three types. In Type I, both parents are heterozygotes, and it is usually caused by a mutation in *SLC3A1.* In non-Type I cystinuria, patients have non-type alleles from both parents, and it is caused by a mutation in *SLC7A9*. Patients with non-Type I cystinuria usually have a variable degree of cystine excretion, and they are not prone to cystine formation. The third type is the mixed type with both Type I and Type II alleles. Due to the lack of genotype–phenotype correlation, it has been advised to define cystinuria according to genetics to Type A, where mutations are found in both *SLC3A1* alleles; Type B, where mutations are found in both *SLC7A9* alleles; and putative type aB, where one mutation is found in each gene (4) and which has a prevalence of 1 in 7000, but there are ethnic and demographic variations (5).

Clinical symptoms usually start around the first two decades of life. Patients with cystinuria have an excessive excretion of cystine in the urine, usually above 400 mg/day (1). Male patients have a more severe clinical presentation with the earlier occurrence and more stones (2, 6). In general, around three-quarters of patients will present with bilateral stones (2, 6, 7). CKD is an important aspect of cystinuria because up to 70% of patients will develop CKD, which can then lead to ESRD (2, 8). Diagnosis is usually made using ultrasound or CT scan, by analyzing the stones that are excreted in the urine, and by the presence of high levels of cystine excretion (1). Genetic testing is not mandatory, but it is recommended as a helpful tool for genetic counseling (9). Treatment is based on dietary advice with adequate hydration, alkalization of urine, and thiol-based therapy, including D-penicillamine and tiopronin, as well as a urological intervention, which includes retrograde ureteroscopy and/or PCNL (1).

Symptomatic nephrolithiasis can complicate 1 in 3300 pregnancies (10), and the incidence of urolithiasis in pregnancy can vary from 1:188 to 1:4600 (11, 12). Having a pregnant patient with cystinuria is challenging for both the nephrologist and the obstetrician. While the impact of cystinuria on pregnancy has not been well studied, it is well known that management is challenging due to regular stone formation and difficult imaging during pregnancy. Therefore, we present an illustrative case of a pregnant patient with cystinuria followed by a multidisciplinary team of urologists, nephrologists, and gynecologists, concluding with a full-term delivery of a healthy child.

## Case description

We present a case of a female patient who was first treated in our hospital at the age of 34 years after an episode of renal colic. Analysis of the expulsed stone showed a cysteine structure. She had a positive family history of chronic kidney disease (CKD) with her brother developing end-stage kidney disease, and she was receiving renal replacement therapy at the age of 28 years. Her medical history revealed an episode of urinary tract infection (UTI) and passing of the urinary stone at the age of 23 years. At the time, she received antibiotic therapy and no further workup was performed. Subsequently, the patient was without any symptoms and had two pregnancies. The first pregnancy had an unremarkable course but her second pregnancy was terminated with an urgent cesarean section in the 39th week with delivery of a healthy child.

**Figure 1:**
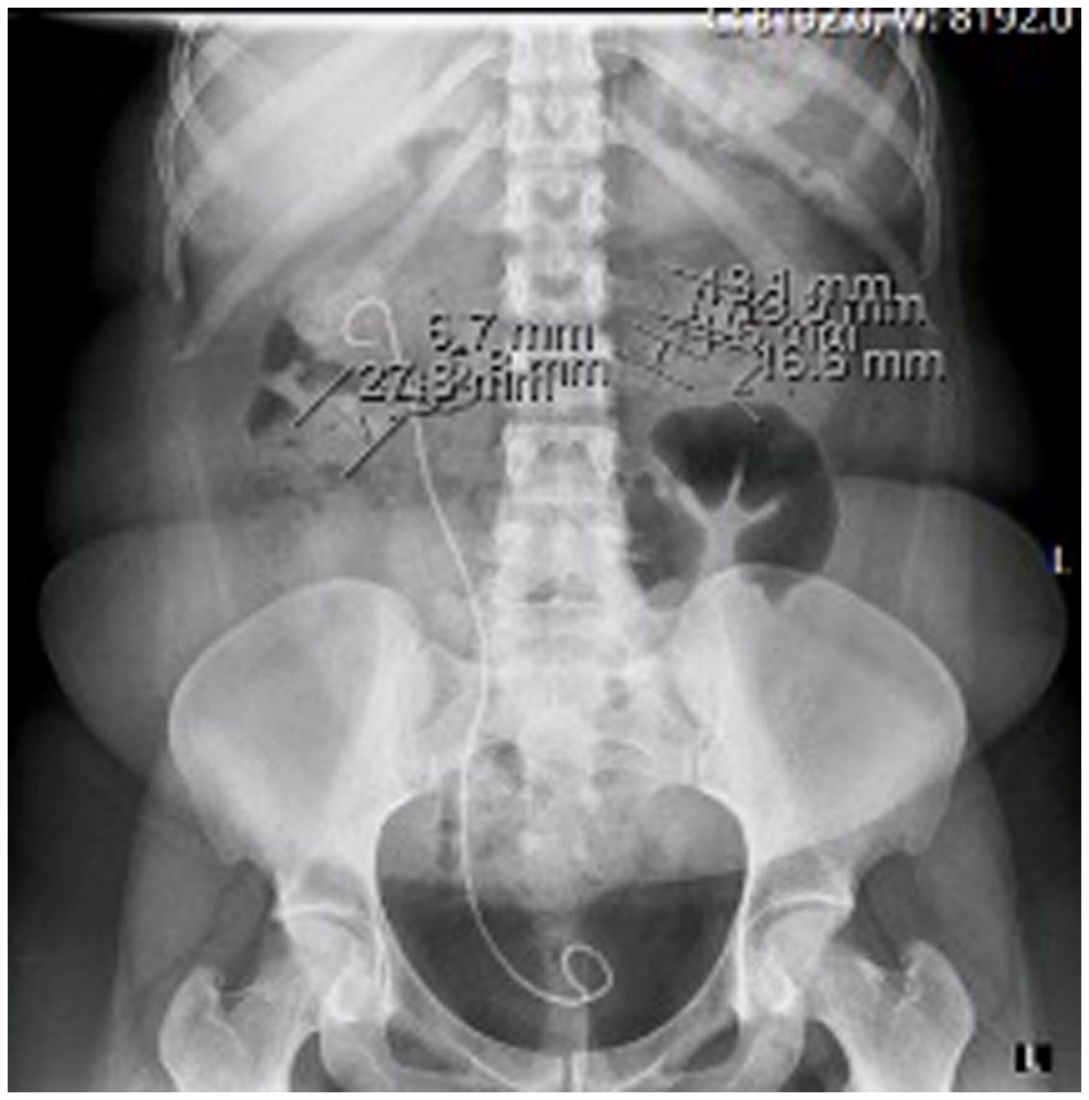
KUB at the time of acute kidney injury.

**Figure 2:**
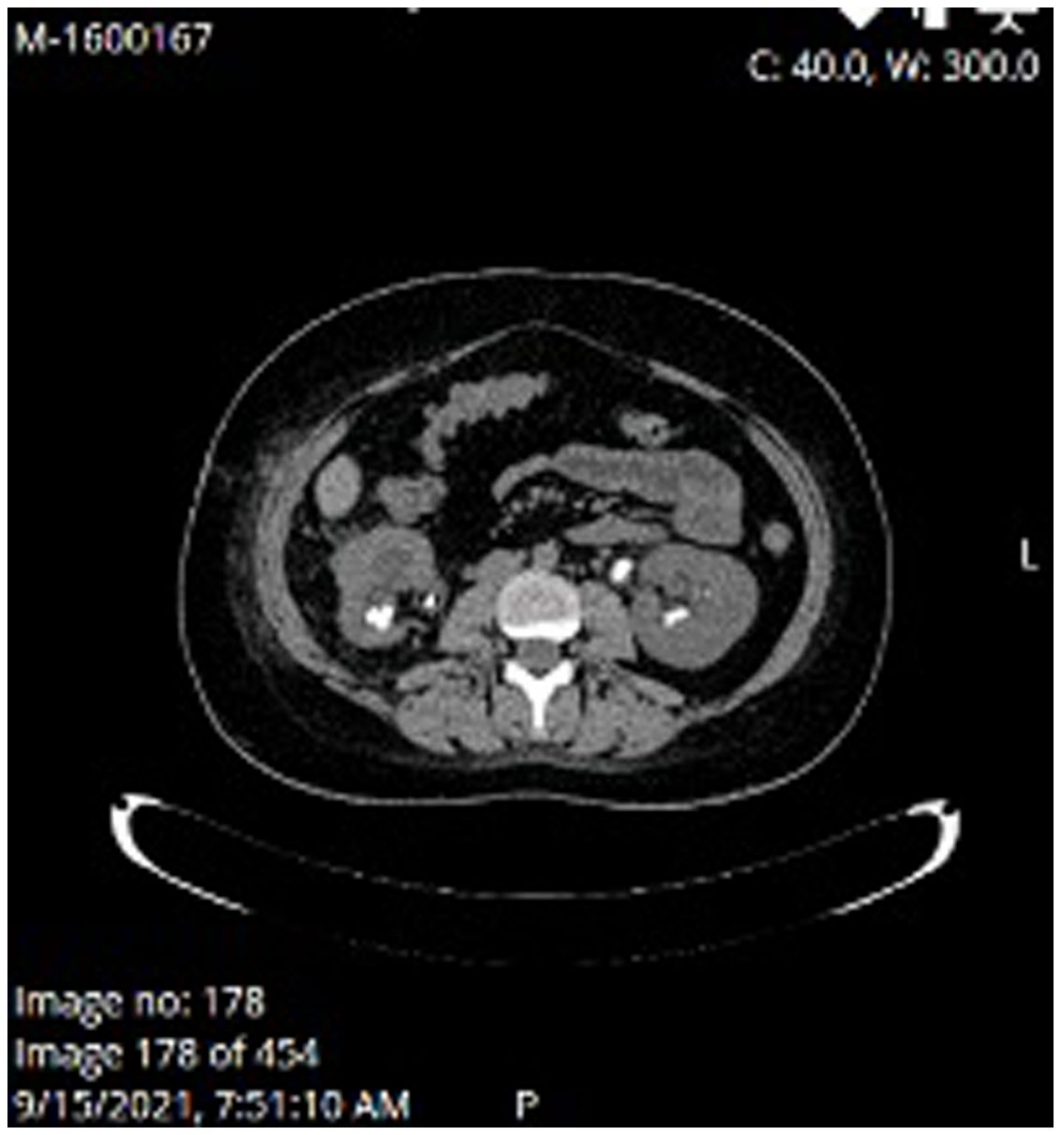
MSCT at the time of acute kidney injury.

At the time of presentation in our hospital, her kidney function was mildly reduced (eGFR 86 mL/min/1.73m^2^) without albuminuria or proteinuria. Ultrasound showed a normal left kidney without signs of lithiasis, while the right kidney had a slightly reduced parenchyma of 10 mm and 3rd degree hydronephrosis. CT scan confirmed hydronephrosis of the right kidney with an impacted stone in the pyelon. Dynamic scintigraphy showed significant functional damage (25%) of the right kidney. After spontaneous expulsion, the stone was sent for analysis that revealed cysteine formations. The case was discussed with the urological team, and in May 2021, a right percutaneous nephrostomy was performed due to obstruction with atrophy of the parenchyma. Dynamic scintigraphy after the procedure showed no improvement in the right kidney function. She was hospitalized in August 2021 for right pyelotomy with ureteral stenting (double J stent) and removal of nephrostomy. One month later she presented with a UTI and worsening kidney function with eGFR of 23 mL/min/1.73m^2^. CT scan showed multiple stones of the left kidney in the lower and middle calyces and cast stones in the lower columns and pyelon. The right kidney was hypotrophic with lobulated contour, signs of post-inflammation changes, and multiple mineral stones along with the previously placed double J stent (Figures 1, 2). Treatment with broad-spectrum antibiotics and hydration was commenced. The right double J stent was replaced and another double J stent was placed in the left kidney. Following treatment, the kidney function started to improve along with a fall in inflammatory markers.

A few weeks later, she was again hospitalized for left percutaneous nephrolithotomy (PCNL), replacement of left double J stent, and left percutaneous nephrostomy. Both double J stents were subsequently removed. At that time, the patient was already pregnant but was not aware of it.

Due to high-risk pregnancy, a multidisciplinary team was assembled, consisting of a nephrologist, urologist, and obstetrician, with scheduled regular monthly check-ups. Her pregnancy was complicated by recurrent UTIs despite prophylaxis with oral fosfomycin and potassium citrate therapy for the alkalinization of urine. Her kidney function remained within normal range and without proteinuria until the 25th week of gestation. At that time, she developed again left renal colic with obstructive nephropathy (grade II hydronephrosis) and acute worsening of kidney function (eGFR 36 mL/min/1,73 m^2^). Under ultrasound guidance, a double J stent was placed again in the left ureter and her kidney function quickly improved to remain normal thereafter, with only slight proteinuria of 0.5–0.6 gram per daily urine (g/d).

In the 38th week of pregnancy, she underwent elective cesarean section delivering a healthy female child with a normal birth weight. The patient was re-evaluated after 3 months. Her kidney function returned to normal (eGFR 82 mL/min/1,73 m^2^) with proteinuria of 0.41 g/d and albuminuria of 0.147 g/d. Cystine values were 111 mmol/mol creatinine (normal level up to 17 mmol/mol creatinine). The treatment with tiopronin was discussed with the patient but, for the time being, the patient opted not to take any medication during lactation.

## Discussion

There are very little data in the literature regarding the management and outcome of pregnancy in patients with cystinuria. Management of urolithiasis in pregnancy has its specific set of problems due to the potential harm that ionizing radiation can have on the developing fetus. The primary diagnostic tool used in a pregnant woman who presents with a clinical picture indicative of ureterolithiasis is an ultrasound of the kidney and bladder. CT scan is contraindicated because of the aforementioned reasons and MR urography is usually unavailable (13).

Due to the physiological stasis of the urine in the kidneys during pregnancy which occurs usually after the 11th week, ultrasound alone does not have sufficient specificity and sensitivity to diagnose ureteral stone in the clinical setting of a potential nephrocolic (14). However, the incidence of urolithiasis does not differ compared to women who are not pregnant (13, 15).

The initial workup of a pregnant female with cystinuria consists of clinical examination and blood workup to exclude uroinfection, while at the same time, analgesic therapy is given to alleviate pain (mostly acetaminophen) (13). Nonsteroidal anti-inflammatory medications should not be given because of adverse consequences for the developing fetus, especially in the first and third trimesters (14). In the case of uroinfection, drainage of the infected and obstructed kidney is necessary. The double J stent and the nephrostomy tube have the same therapeutic effect. Due to the rapid development of encrustations, there is a need for regular replacements of either the double J stent or the nephrostomy tube until delivery (13, 14). In some tertiary centers, ureteroscopy is a therapeutic option, especially in the central trimester, and when done by an experienced endourologist (16). Fortunately, in more than 80% of cases, conservative treatment leads to spontaneous expulsion of the stone without the need for further interventions (17).

Pregnant women with cystinuria who are hospitalized for nephrolithiasis have a higher risk of preterm delivery and pyelonephritis (18). Gregory et al. (18) published an article describing 46 pregnancies in patients with cystinuria who were treated with a high fluid intake alone or in combination with D-penicillamine. Of 46 pregnancies, 41 resulted in live births of normal children, four had spontaneous abortions, and one was terminated due to concerns for the mother’s health. These authors reported that in 18 patients, new calculi appeared. Renal colic appeared in seven patients and two patients passed stones. In our patient, stones were not excreted in urine during pregnancy, but she had several renal colic attacks which presented with acute worsening of kidney function, and this led to hospitalization, antibiotic treatment, and urological interventions. Although hypertension is a common complication of cystinuria (1), our patient had normal blood pressure during the entire pregnancy.

Thiol-based drugs in pregnancy have been used according to literature and published case reports. FDA is advising against the use of tiopronin in pregnancy (19), but available data do not reveal significant drug-associated risk for adverse maternal or fetal outcomes, miscarriage, or major birth defects. Tiopronin and metabolites are not excreted in human milk nor were found in the serum of children whose mothers were taking tiopronin during lactation (20). Nevertheless, it is not recommended during lactation due to other adverse effects such as nephrotic syndrome. Furthermore, tiopronin can induce a significant reduction in prolactin and suppress lactation (20). D-penicillamine has FDA pregnancy categorization D, which means that there is evidence of human fetal risk, but in some cases, potential benefits can outweigh the risk (21).

Shee and Pais Jr. published a case report of a pregnant patient who presented with flank pain and moderate hydroureter seen on ultrasound, treated with nephrostomy tube placement during pregnancy, and after full-term cesarean section, the patient underwent PCNL that revealed cystine stone as the culprit (22). As opposed to Shee and Pais Jr. (22), although presenting with similar symptoms, our patient already had complications and urological interventions during early pregnancy, as well as later. Unlike the majority of patients with cystinuria, of which around 70% have CKD, our patient had stable kidney function at the time of diagnosis, despite the hypofunction of one kidney. The brother of our patient was already treated with renal replacement therapy at the age of 28 years and a major concern, in this case, was the possibility of deterioration of kidney function during pregnancy. Despite a complicated course of pregnancy, her kidney function deteriorated only as a consequence of obstruction with quick improvement after urological intervention.

It is important to emphasize the importance of a multidisciplinary approach in these specific cases. In our case, the patient was regularly seen by a nephrologist, urologist, and obstetrician and was monitored for potential complications, and treated adequately when they occurred. The patient had a successful pregnancy in the end, but her pregnancy course was complicated, so it is important to advise female patients with cystinuria of available methods of contraception, especially if they already had children, to avoid possible negative outcomes.

## Conclusion – patient perspective

Reports on pregnancy with cystinuria are scarce in the literature. Pregnancy leads to several physiological changes, including changes in the urine content of metabolites. Ultrasound is a readily available tool and should be used as a standard of care in all pregnancies which will aid in the early diagnosis of urolithiasis. If ultrasound had been done in the first two pregnancies, our patient would probably have been diagnosed with cystinuria earlier and very probably hypofunction of the right kidney could have been prevented. This case shows that even patients with complex medical problems and complicated disease courses during pregnancy can still have a successful perinatal outcome.

## Data availability statement

The raw data supporting the conclusions of this article will be made available by the authors, without undue reservation.

## Ethics statement

Ethical review and approval was not required for the study on human participants in accordance with the local legislation and institutional requirements. Written informed consent from the patients/participants was not required to participate in this study in accordance with the national legislation and the institutional requirements. Written informed consent was obtained from the patient to publish this case report, including all data and images.

## Author contributions

IE has analysed data and literature about the article and made design and conception of this article. MM has equally contributed in revision of this manuscript with advices about urological clinical course. E-GV has equally contributed in revision of the manuscript and gave advices about gyaenocological clinical course. FPM has equally contributed in revision of the manuscript and gave perspective and advices from nephrologist perspective. LL has equally contributed in revision of the manuscript and gave advices from paediatrician perspective. JB has equally contributed in this article and gave advices for nephrologist perspective. VI has equally contributed in revision of this manuscript and helped in critical revision. All of the authors have read the manuscript, attest to the validity and legitimacy of the data and its interpretation, and agree to its submission to Frontiers in Medicine. All authors had equally participated in final approval of the article, administrative, technical or logistical support.

## Conflict of interest

The authors declare that the research was conducted in the absence of any commercial or financial relationships that could be construed as a potential conflict of interest.

## Publisher’s note

All claims expressed in this article are solely those of the authors and do not necessarily represent those of their affiliated organizations, or those of the publisher, the editors and the reviewers. Any product that may be evaluated in this article, or claim that may be made by its manufacturer, is not guaranteed or endorsed by the publisher.
